# Analysis of Eradication, Recurrence and Levels of 25-hydroxyvitamin D_3_ and Interleukin-1β in paediatric patients with Helicobacter Pylori Infection-related Gastritis

**DOI:** 10.12669/pjms.36.6.2292

**Published:** 2020

**Authors:** Yuanda Zhang, Binbin Bi, Xu Guo, Shaohui Zhang

**Affiliations:** 1 Yuanda Zhang, Department of Gastroenterology, Baoding Children’s Hospital, Baoding, Hebei, 071000, P.R. China; Key Laboratory for Clinical Research of Respiratory and Digestive Diseases in Children, Baoding, Hebei, 071000, China; 2 Binbin Bi, Department of Gastroenterology, Baoding Children’s Hospital, Baoding, Hebei, 071000, P.R. China; Key Laboratory for Clinical Research of Respiratory and Digestive Diseases in Children, Baoding, Hebei, 071000, China; 3 Xu Guo, Department of Gastroenterology, Baoding Children’s Hospital, Baoding, Hebei, 071000, P.R. China; Key Laboratory for Clinical Research of Respiratory and Digestive Diseases in Children, Baoding, Hebei, 071000, China; 4 Shaohui Zhang, Department of Gastroenterology, Baoding Children’s Hospital, Baoding, Hebei, 071000, P.R. China; Key Laboratory for Clinical Research of Respiratory and Digestive Diseases in Children, Baoding, Hebei, 071000, China

**Keywords:** Helicobacter Pylori (Hp), 25-(OH) D_3_, IL-1β, Children, Eradiation, Recurrence

## Abstract

**Objective:**

To study whether the levels of 25-hydroxyvitamin D_3_ (25-(OH)D_3_) and Interleukin-1β (IL-1β) are correlated to the eradication and recurrence of helicobacter pylori (Hp) in paediatric patients with Hp infection-related gastritis.

**Methods:**

A total of 142 paediatric patients with Hp infection-related gastritis from November 2017 to March 2018 in Baoding Children’s Hospital were selected as study subjectswere selected as study subjects and were followed up for one year. Paediatric patients were grouped and analyzed according to the effect of follow-up treatment and recurrence.

**Results:**

The levels of 25-(OH) D_3_ in the non-eradication group were lower than those in the eradication group and the control group (F=27.087, P<0.05); the levels of IL-1β were higher than those in the eradication group and the control group (F=16.610, P<0.05). Recurrence during follow-up visits: The levels of 25-(OH) D_3_ in the recurrence group were lower than those in the non-recurrence group and the control group (F=33.837, P<0.05); the levels of IL-1β in the recurrence group were higher than those in the non-recurrence group and the control group (F=7.896, P<0.05). Correlation analysis showed the levels of 25-(OH) D_3_ and IL-1β in the eradication group and the non-eradication group were negatively correlated (r=-0.232, P<0.05); the levels of 25-(OH) D_3_ and IL-1β in the recurrence group and the non-recurrence group were negatively correlated (r=-0.225, P<0.05).

**Conclusion:**

Relatively high levels of IL-1β may be correlated to the difficulty in eradicating the Hp infection in paediatric patients. Relatively low levels of 25-(OH) D_3_ may be correlated to the difficulty in eradicating the Hp infection and recurrence in paediatric patients.

## INTRODUCTION

Helicobacter pylori (Hp) infection is a main cause of chronic gastritis and duodenal ulcer in children.^1^ The incidence of Hp infection in children in China is high; and its natural remission rate is low.^2^ In recent years, the eradication rate of Hp infection has decreased. The eradication rate of Hp infection treated by triple therapy has been reported to be lower than 80%.^3^ In addition, patients with Hp infection partially eradicated may have recurrence. Vitamin D (VitD) has anti-inflammatory and immunomodulatory effects. The decrease of VitD levels may increase disease susceptibility. VitD deficiency has been reported to increase the risk of Hp infection.^4^ The detection of 25-hydroxyvitamin D_3_ (25-(OH)D_3_) can reflect the VitD levels in the human body. Interleukin-1β (IL-1β) has been reported to be related to Hp infection. IL-1β can adjust the pH level in the stomach, thus affecting the outcome of Hp treatment.^5^ In order to understand whether the levels of VitD and IL-1β are correlated to the eradication and recurrence in paediatric patients with Hp infection-related gastritis, the levels of 25-(OH)D_3_ and IL-1β in paediatric patients with Hp infection were detected in this study; and these patients were followed up for one year.

## METHODS

One hundred forty two paediatric patients (95.9%) successfully followed up for one year among the 148 paediatric patients with Hp infection-related gastritis treated in Baoding Children’s Hospital from November 2017 to March 2018 were selected as study subjects, including 81 male and 61 female patients with an average age of 9.0±2.2. All paediatric patients were treated with amoxicillin + clarithromycin + omeprazole for two weeks. The C^13^ breath test was re-examined four weeks after stopping using the drug. Paediatric patients with negative results of the re-examination of C^13^ breath test were followed up for one year. The C^13^ breath test was carried out again half a year and one year after the results of C^13^ breath test turned negative. Sample collection 4ml of fasting venous blood was drawn in the morning before the treatment upon the first visit by the enrolled paediatric patients with Hp infection-related gastritis. 4ml fasting venous blood of healthy children was reserved in the morning.

### Ethical Approval

The study was approved by the Institutional Ethics Committee of Baoding Children’s Hospital, (Dated: January 15, 2019) and written informed consents were obtained from all participants

### Exclusion criteria

1. Paediatric patients who had a history of severe liver, heart and kidney diseases and rickets;
2. Paediatric patients who had been administrated with VitD or hormone within half a year;
3. Paediatric patients who had poor compliance and could not complete regular follow-up visits.

The diagnostic criteria proposed by European Society for Paediatric Gastroenterology, Hepatology, and Nutrition, ESPGHAN/North American society for pediatric gastroenterology, Hepatology, and nutrition, NASPGHAN, were adopted in this study.^6^ All paediatric patients enrolled had gastrointestinal symptoms (such as abdominal pain and vomiting); paediatric patients with positive results of C^13^ breath test and/or rapid urease test and endoscopic pathological staining were diagnosed with Hp infection. The levels of 25-(OH) D_3_ were determined by the Key Laboratory of Respiratory and Digestive Diseases in Children of Baoding, Hebei Province, with Abbott i2000 chemiluminescence instrument by chemiluminescence method. The kit was purchased from Abbott. The test was carried out by a specially-assigned person.Detection of IL-1β The levels of IL-1β was detected by Zhongtong Lanbo Clinical Laboratory Co., Ltd. by euzymelinked immunosorbent assay (ELISA). The kit was purchased from Shanghai Fanke Biotechnology Co., Ltd.SPSS22.0 statistical software package was used for the statistical analysis in this study. The measurement data were expressed as (). T test and variance analysis were used for the comparison among groups. The difference was statistically significant for P ≤ 0.05.

## RESULTS

Conditions of the control group: 62 healthy children who had received physical examination in our hospital during the same period were selected as a control group, including 34 male and 28 female patients with an average age of 9.1±2.3. After the treatment of 142 patients with Hp infection-related gastritis, 106 patients (74.6%) with a negative result of the reexamination by C^13^ breath test 4 weeks after stopping using the drug formed an eradication group, including 59 male and 47 female patients with an average age of 9.1±2.2. Thirty-six patients (25.4%) with a positive result formed a non-eradication group, including 22 male and 14 female patients with an average age of 9.0±2.3. The difference in age and gender among the eradication group, the non-eradication group and the control group had no statistical significance (P= 0.997, P=0.815).

Among the 106 paediatric patients with the disease eradicated, 22 patients (20.8%) who had positive result of re-examination by C^13^ breath test again during the 1-year follow-up visit formed a recurrence group, including 12 male and 10 female patients with an average age of 9.0±2.1; 84 paediatric patients (79.2%) who had a negative result formed a non-recurrence group, including 47 male and 37 female patients with an average age of 9.1±2.2. The difference in age and gender among the recurrence group, the non-recurrence group and the control group had no statistical significance (P=0.970, P=0.988).

Analysis of levels of 25-(OH) D_3_ and IL-1β in the eradication group, the non-eradication group and the control group The levels of 25-(OH) D_3_ in the non-eradication group were lower than those in the eradication group and the control group (F=27.087, P=0.000); the levels of 25-(OH) D_3_ in the non-eradication group were lower than those in the eradication group (t=2.215, P=0.033); the levels of IL-1β were higher than those in the eradication group and the control group (F=16.610, P=0.000); the levels of IL-1β in the non-eradication group were higher than those in the eradication group (t=3.299, P=0.002). Table-I

**Table-I:** Comparison of Levels of 25-(OH) D_3_ among the Eradication Group, the Non-eradication Group and the Control Group.

Group	Number of cases	25-(OH) D3(ug/L)	IL-1β(ug/L)
Eradication group	106	27.1±6.0	281.2±95.3
Non-eradication group	36	24.6±5.4	343.5±105.4
Control group	62	32.1±3.9	233.7±74.2
F value		27.087	16.610
P value		0.000	0.000

Analysis of levels of 25-(OH) D_3_ and IL-1β in the recurrence group, the non-recurrence group and the control group The levels of 25-(OH) D_3_ in the recurrence group were lower than those in the non-recurrence group and the control group (F=33.837, P=0.000); the levels of 25-(OH) D_3_ in the recurrence group were lower than those in the non-recurrence group, and the difference had statistical significance (t=4.755, P=0.000); the levels of IL-1β in the recurrence group were higher than those in the non-recurrence group and the control group (F=7.896, P=0.001); the difference in the levels of IL-1β between the recurrence group and the non-recurrence group had no statistical significance (t=1.901, P=0.06). Table-II

**Table-II:** Comparison of Levels of 25-(OH) D_3_ among the Recurrence Group, the Non-recurrence Group and the Control Group.

Group	Number of cases	25-(OH) D_3_ (ug/L)	IL-1β(ug/L)
Recurrence group	22	22.1±4.9	315.2±100.7
Non-recurrence group	84	28.4±5.6	272.3±92.5
Control group	62	32.1±3.9	233.7±74.2
F value		33.837	7.896
P value		0.000	0.001

### Correlation Analysis

The levels of 25-(OH) D_3_ and IL-1β in the eradication group and the non-eradication group were negatively correlated (r=-0.232, P<0.05). The levels of 25-(OH) D_3_ and IL-1β in the recurrence group and the non-recurrence group were negatively correlated (r=-0.225, P<0.05).

## DISCUSSION

Helicobacter pylori (Hp) is a common human pathogen. The triple therapy of amoxicillin + clarithromycin + proton pump inhibitor (PPI) is often used to eradicate Hp in Children. Currently, the eradication rate of the first-line triple therapy in most regions is below 80%.^3^ The local eradication rate is about 70%.^7^ This study showed that the eradication rate of the first-line triple therapy in children in this region is 74.6%, which is consistent with the above statements. Some paediatric patients with Hp infection may have recurrence, but there are great regional and age differences. The recurrence rate in economically underdeveloped regions and children is high.^8^,^9^ One year after the eradication, the recurrence rate was reported to be 6% in Pakistan,^10^ 20% in children below 5 years old, 20% in 5-9-year-old children and 8% in children above 10 in Bolivia.^9^ The recurrence rate of Hp infection one year after the eradication in urban population in China is relatively low, only 1.75%.^11^ This study showed that the 1-year recurrence rate in Children in this region was as high as 20.8%, which was considered to be correlated to the young age for all patients enrolled in this study were children and the relatively backward economy in this region.

The levels of VitD can change the risk of Hp infection.^12^ Several studies have shown that the deficiency of 25-(OH)D3 is negatively correlated to the eradication of Hp.^13^-^15^ The results of this study showed that the levels of 25-(OH)D_3_ are lower than those in healthy paediatric patients. In addition, the levels in the non-eradication group are lower than those in the eradication group. This is consistent with the above statements and supports the viewpoint that the relatively low levels of 25-(OH)D_3_ are one of the risk factors of Hp infection and failure of eradication. Moreover, this study also showed that the levels of 25-(OH)D_3_ in the recurrence group were lower than those in the non-recurrence group, indicating that the lower levels of 25-(OH)D_3_ in children with Hp infection, the higher the 1-year recurrence rate after eradication.

25-(OH)D_3_ may affect Hp infection by the following ways: 1. VitD can activate the autosomal degradation of Hp through the PDIA3 receptor in gastric epithelial cells, driving the elimination of Hp through the autosomal pathway^16^; 2. The decomposition product VDP1 derived from VitD^3^ has antibacterial effect on Hp^17^; 3. The reduction of VitD receptor induces the generation of CAMP, and increases the expression levels of IL-6 and IL8 / CXCL8.^18^

Previous studies have suggested that Hp infection can induce the increase of the IL-1β levels.^19^,^20^ Besides, high levels of IL-1β are closely correlated to Hp infection-related gastritis.^20^ According to this study, the IL-1β levels in paediatric patients with Hp infection were higher than those in healthy children, indicating that paediatric patients with Hp infection may have elevated IL-1β levels. Meanwhile, this study showed that the IL-1β levels in the non-eradication group were higher than those in the eradication group, indicating that the relatively high IL-1β levels may be one of the reasons for the difficulty in the eradication of Hp infection in children. In this study, the difference in the IL-1β levels between the recurrence group and the non-recurrence group had no statistical significance, indicating that the changes in the IL-1β levels are not correlated to the recurrence rate of Hp infection.

VitD can regulate inflammatory cytokines.^21^ This study showed that the levels of 25-(OH) D_3_ and IL-1β in paediatric patients with Hp infection were negatively correlated. Children with Hp infection-related gastritis were considered to have relatively high IL-1β levels and relatively low 25-(OH) D_3_ levels due to chronic inflammatory reactions. However, the decrease of 25-(OH) D_3_ levels lowered the levels of inflammatory cytokines. The two were involved in the process of Hp infection.

### Limitations of the study

The present study is a single-center study, sample size and short follow-up period. Therefore, the plan for future research would to be increase the sample size and extend the follow-up time. For future studies, the relationship between Hp eradication recurrence and 25-(OH) D_3_-as well as IL-1β would be further analyzed.

## CONCLUSION

In conclusion, 25-(OH) D_3_ and IL-1β can affect Hp infection in multiple ways. The relatively high levels of IL-1β may be correlated to the difficulty in the eradication of Hp infection, while the low levels of 25-(OH) D_3_ may be relevant to the difficulty in the eradication of Hp infection and the recurrence.

### Authors’ Contributions:

**YZ and** **SZ:** Designed this study and prepared this manuscript, and responsible for integrity of research among the listed authors.

**XG & BB:** Collected and analyzed clinical data;

**LT:** Significantly revised this manuscript.
